# High Concentrations of Serum Soluble E-Cadherin in Patients With Q Fever

**DOI:** 10.3389/fcimb.2019.00219

**Published:** 2019-06-21

**Authors:** Soraya Mezouar, Ikram Omar Osman, Cléa Melenotte, Camélia Slimani, Céline Chartier, Didier Raoult, Jean-Louis Mege, Christian A. Devaux

**Affiliations:** ^1^Aix-Marseille Univ, IRD, APHM, MEPHI, IHU-Méditerranée Infection, Marseille, France; ^2^APHM, IHU-Méditerranée Infection, UF Immunologie, Marseille, France; ^3^CNRS, Marseille, France

**Keywords:** Q fever, *coxiella burnetii*, adhesion molecule, soluble E-cadherin, biomarker

## Abstract

Cadherins switching is a hallmark of neoplasic processes. The E-cadherin (E-cad) subtype is one of the surface molecules regulating cell-to-cell adhesion. After its cleavage by sheddases, a soluble fragment (sE-cad) is released that has been identified as a pro-carcinogenic inflammatory signal in several bacteria-induced cancers. Recently we reported that Q fever, a disease due to *Coxiella burnetii* infection, can be complicated by occurrence of non-Hodgkin lymphoma (NHL). Therefore, we studied E-cad switching in Q fever. The sE-cad levels were found increased in the sera of acute and persistent Q fever patients, whereas they remained at the baseline in controls groups of healthy donors, people cured of Q fever, patients suffering from unrelated inflammatory diseases, and past Q fever patients who developed NHL. These results indicate that sE-cad can be considered as a new biomarker of *C. burnetii* infection rather than a marker of NHL-associated to Q fever. We wondered if changes in sE-cad reflected variations in the *CDH1* gene transcription. The expression of E-cad mRNA and its intracellular ligand β-catenin was down-regulated in peripheral blood mononuclear cells (PBMCs) of patients with either acute or persistent forms of Q fever. Indeed, a lower cell-surface expression of E-cad was measured in a minority (<5%) subpopulation of HLADR^+^/CD16^+^ monocytes from patients with acute Q fever. However, a very strong increase in E-cad expression was observed on more than 30% of the HLADR^+^/CD16^+^ monocytes of persistent Q fever patients, a cell subpopulation known to be a target for *C. burnetii* in humans. An experimental *in vitro* infection of healthy donors' PBMCs with *C. burnetii*, was performed to directly evaluate the link between *C. burnetii* interaction with PBMCs and their E-cad expression. A significant increase in the percentage of HLADR^+^/CD16^+^ monocytes expressing E-cad was measured after PBMCs had been incubated for 8 h with *C. burnetii* Nine Mile strain. Altogether, these data demonstrate that *C. burnetii* severely impairs the E-cad expression in circulating cells of Q fever patients.

## Introduction

Q fever is a zoonosis most of the time transmitted to humans by animal products contaminated with *Coxiella burnetii*, a strict intracellular Gram-negative bacterium (Maurin and Raoult, 1999). The bacteria usually infect human by the aerosol route and Q fever usually occurs 2–6 weeks after exposure (van Schaik et al., 2013). In humans, the infection most often remains asymptomatic. Symptomatic infections (10–60% of cases), usually resolve spontaneously in a few weeks. This clinical form of the disease, called acute Q fever, is characterized by high fever, headache, myalgia, pneumonia, and hepatitis (Eldin et al., 2017). In less than 5% of cases, the symptoms do not resolve and settle in a persistent way (Melenotte et al., 2018). The patients suffering from Q fever can therefore be stratified into different categories ranging from acute Q fever to persistent Q fever with or without lymphadenitis in which the bacterium is detectable (Tattevin et al., 2003; Melenotte et al., 2016). The infection can evolve toward endocarditis, vascular infection, osteoarticular infection (Million et al., 2013; Melenotte et al., 2018). Other disorders were also described in association with persistent focalized *C. burnetii* infections, including interstitial lung diseases, persistent granulomatous hepatitis, and B-cell lymphoma (Ramos et al., 2012; Melenotte et al., 2016, 2018).

So far, the nature of factors that determine the severity of Q fever remain mostly unknown (Deng et al., 2012; Shipman et al., 2013; Stead et al., 2013; Martinez et al., 2014; Schoffelen et al., 2017). Recently, we have reported that the presence of circulating anticardiolipin antibodies during the acute phase of the disease was associated with acute Q fever complications, such as endocarditis, cholecystic, and hemophagocytic syndrome (Melenotte et al., 2018). Until recently, the molecular mechanisms that may account for the progression of Q fever toward lymphoma were largely ignored. Thereby, we investigated the transcriptional signature associated with the development of NHL using the PBMCs of patients with Q fever. By analyzing their gene expression by microarray, we found the over-expression of genes involved in anti-apoptotic process and repression of pro-apoptotic genes as compared to samples from healthy donors (Melenotte et al., 2019). Yet, the early molecular events controlling the balance between remission and progression toward more severe diseases remain elusive.

There is accumulating evidence reported in the infectious diseases literature indicating that bacterial invasions of human hosts and transmigration at the epithelium level involves the over-expression of sheddases which mediate the degradation of cell surface adhesion molecules (CAM). The cleavage of CAM leads to cellular tissue destruction and rapid spread of bacteria (Elkington et al., 2005; Vanlaere and Libert, 2009). The matrix metalloproteinases (MMP) family, count among the inducible human sheddases that catalyze the proteolysis of cadherins (cad), a group of cell-surface adhesion molecules that includes E-cadherin (E-cad) and belongs to the superfamily of CAM (Grabowska and Day, 2012). Recently, we reported (Jansen et al., 2017) that *C. burnetii* induces the sheddases MMP-1 and MMP-9 production in PBMCs of healthy persons exposed *in vitro* to the bacterium; that the single nucleotide polymorphisms (SNPs) in MMP-7 and MMP-9 were more frequent in patients with persistent Q fever; and, that the circulating MMP-7 serum levels were higher in patients with persistent Q fever.

The aim of this study was to focus our attention on the modulation of E-cad expression in patients with Q fever. The modulation of E-cad cell-surface expression and the release of a soluble form of E-cad (sE-cad) have been identified as biomarkers during bacterial invasion of the human host and as a trigger in neoplasic processes induced by several bacterial species. E-cad cleavage has already been associated with breast, gastric and colorectal solid tumors (Repetto et al., 2014), and multiple myeloma induction (Syrigos et al., 2004). It was reported in the literature that *Helicobacter pylori*, a risk factor for the development of gastric adenocarcinoma, modulates the expression of the E-cad coding gene *CDH1* (Jacobs et al., 2011), and activates the calpain sheddase, generating sE-cad and inducing a relocalization of β-catenin (β-cat), an E-cad intracellular ligand (O'Connor et al., 2011). It was also documented that the *Bacteroides fragilis toxin*, a known bacterial sheddase, coordinates a pro-carcinogenic inflammatory cascade via the release of sE-cad from colonic epithelial cells, which triggers myeloid cell-dependent distal colon tumorigenesis (Wu et al., 2003; Chung et al., 2018). In addition, colorectal tumor development linked to *Streptococcus gallolyticus* was found to correlate with an increase in β-cat expression (Kumar et al., 2017). Moreover, phosphorylation of β-cat by AKT promotes the transcriptional activity of the β-cat gene (Fang et al., 2007). Finally, increase in β-cat has been reported in NHL (Ge et al., 2012). Because the cleavage of E-cad and modulation of E-cad/ β-cat complex expression seem to be a target for several bacteria-inducing cancers, we investigated whether a similar mechanism might occur in Q fever, possibly accounting for shaping a microenvironment favorable to the initiation of NHL.

Herein, we evidenced for the first time that sE-cad is released from cell membrane in patients with Q fever, and that *C. burnetii* infection modulates the expression of the *CDH-1*/E-cadherin and *CTNNB1*/β-catenin genes and the cell-surface expression of E-cad on monocytes during Q fever.

## Materials and Methods

### Patients Population

We included 38 patients followed in the French National reference center for Q fever in Marseille France (“Centre National de Référence-CNR des Rickettsia, Coxiella, et bartonella”). Samples were collected from patients with acute Q fever, persistent focalized *C. burnetii* infection, and persistent focalized *C. burnetii* lymphadenitis or lymphoma. Acute Q fever was defined by the association of clinical symptom (fever and/or hepatitis and/or pneumonia and/or lymphadenitis) with serologic criteria for phase II IgG levels ≥200 and phase II IgM ≥50 levels within the 3 months after the onset of symptoms. The persistence of symptoms for more than 3 months in addition to the identification of an infectious focus and to positive serology were the three criteria requested to diagnose persistent focalized *C. burnetii* infection (Eldin et al., 2017; Melenotte et al., 2018). Clinical characteristics of Q fever patients included are summarized in Table 1. It must be emphasized that for reasons of availability of samples that meet the methodological constraints of the assays (i.e., for the sE-cad ELISA, plasma should be collected using heparin as an anticoagulant; EDTA plasma is not suitable for use in this assay due to its chelating properties), it is not systematically the same patients who have been tested by flow cytometry, qRT-PCR, and ELISA. Indeed, samples from 11 patients were tested by two or three assays (samples from nine patients were tested by flow cytometry and qRT-PCR; samples from five patients were tested by flow cytometry and ELISA; samples from three patients were tested using the three different assays). Healthy donors were used as control and samples were obtained from the national blood transfusion center (“Etablissement Français du Sang,” EFS, Marseille, France). For the ELISA, in addition to the Q fever patients and healthy donors we also included 10 people cured of Q fever, 5 acute (3 men, 2 women) and 5 persistent (all men) with IgG levels ≥800 (people followed in the French National reference center for Q fever, Marseille, France), and 10 patients (9 women, 1 man) suffering from rheumatoid arthritis (RA) and diagnosed according to the 2010 ACR/EULAR criteria (Aletaha et al., 2010). The mean age of the cured patients was 67 ± 6.7 and 49.8 ± 14.9 for persistent and acute Q fever forms, respectively mean ± standard deviation). The mean age of the RA patient was 60 ± 12.7 years (mean ± standard deviation) with disease duration of 15.2 ± 9.3 years. Informed written consent was obtained from each subject with the approbation of the ethical committee of the IHU Méditerranée-Infection n°2016-024.

**Table 1:** Patients with *C. burnetii* infection characteristics.

**Patient**	**Flow cytometry *n* = 10**	**q-RTPCR *n* = 29**	**ELISA *n* = 26**
Sex Men/Women	8/2	21/8	22/4
Mean age (±SD)	64.1 ± 12.6	56 ± 14.5	54.1 ± 20.7
**Acute Q fever**			
***n*=**	**6**	**14**	**11**
Pneumonia only	2	0	4
Hepatitis only	3	12	7
Pneumonia and hepatitis	1	2	0
**Persistent** ***C. burnetii*** **infection**			
***n*****=**	**4**	**15**	**15**
Endocarditis	4	14	1
Vascular infection	0	1	3
Osteoarticular infection	0	0	1
Hepatitis only	0	0	1
Lymphadenitis	0	0	4
Lymphadenitis and hepatitis	0	0	3
Lymphadenitis and pneumonia	0	0	0
Lymphadenitis and lymphoma	0	0	2
**Treatment DP**			
Before treatment	10	4	14
During treatment	0	13	12
After treatment	0	12	10

*Bold values represent the total patients included for each groups*.

### Cell Isolation

PBMCs from healthy donors or patients were recovered from samples collected on leukopacks from “Etablissement français du sang” or Ethylenediaminetetraacetate (EDTA) tubes (Sigma Aldrich, Saint-Quentin Fallavier, France), respectively using Ficoll (Eurobio, Les Ulis, France) density gradient centrifugation as previously described (Ka et al., 2016).

### Bacteria Production and *in vitro* Infection of PBMCs

*Coxiella burnetii* Nine Mile (RSA496) strain cultured on L929 cells for 8 days were harvested from culture supernatant after cells had been sonicated and centrifuged, as previously described (Ka et al., 2016). *C. burnetii* present in cell-free supernatants were collected, centrifuged at 10,000 x g for 10 min, washed and stored at −80°C until they were used. For the PBMC infection experiments, 10^7^ live bacteria (ratio 1:1) were incubated for 8 h with 10^7^ PBMCs. As controls, PBMCs were either incubated for 8 h in bacteria-free cell-culture medium or in culture medium supplemented with 10 ng/ml of lipopolysaccharide (LPS) from *Escherichia coli* (Sigma Aldrich, Saint-Quentin Fallavier, France).

### Ribonucleic Acid (RNA) Isolation

RNAs were extracted from whole blood or cells using PAXgene blood RNA kit or RNeasy Mini Kit, respectively. This extraction process includes a DNase I treatment to eliminate DNA contaminants according to the manufacturer instructions (Qiagen, Courtaboeuf, France) as previously described (Thuny et al., 2012).

### Quantitative-Reverse Transcription-Polymerase Chain Reaction (qRT-PCR)

qRT-PCR was performed to obtain cDNA using the Moloney murine leukemia virus-reverse transcriptase (MMLV-RT) kit (Life Technologies) as previously described (Belhareth et al., 2018). qRT-PCR was realized using the SYBR Green Fast Master Mix (Roche Diagnostics, Meylan, France) and specific primers (designed using the Primer3 software).We used primers for *CDH1* gene (forward: gaaggtgacagagcctctggat and reverse: gatcggttaccgtgatcaaaat) coding for the E-cad mRNA; *CTNNB1* gene (forward: agcttccagacacgctatcat and reverse: cggtacaacgagctgtttctac) coding for the β-cat mRNA; and to the housekeeping gene β-actin (forward: aggaaggaaggctggaagag and reverse: ggaaatcgtgcgtgacatta). The qRT-PCR results obtained using the *CDH1* and *CTNNB1* probes were normalized to β-actin. Data were expressed as Log10 (2^ΔCt^), where ΔCt = (Ct_Target_−Ct_Actin_). The threshold cycle (Ct) was defined as the number of cycles required to detect the fluorescent signal.

### Soluble E-Cadherin Quantification

Sera from acute Q fever, persistent Q fever, rheumatoid arthritis patients and healthy controls were isolated from dry tubes. The quantity of soluble E-cad protein concentration in the patients' sera was determined using an immunoassay kit according to the manufacturer's instructions (R&D system, Lille, France). The minimal detectable concentration of human E-cad is 0.039 ng/ml.

### Flow Cytometry

The membrane expression of E-cad on PBMCs was investigated using flow cytometry. Cells were analyzed according to fluorescence intensity for CD3, CD14, CD16, and CD20, markers. MAb anti-CD3-FITC, anti-CD20-PC5, anti-CD16-PE, and anti-CD14-FITC were purchased from Beckman (Beckman coulter, Villepinte, France). Cell surface expression of E-cad was assayed using an anti-E-cad mAb-APC. Fluorescence intensity was measured using a Canto II cytofluorometer (Becton Dickinson, Biosciences, Le Pont de Claix, France), and the results were analyzed using a FlowJo software X.10.0.7.

### Statistical Analysis

A two-by-two comparison of qRT-PCR data was performed using a two-tailed non-parametric Mann–Whitney *U*-test. All tests were 2-sided and *p* < 0.05 was considered significant.

## Results

### Increase of Soluble E-Cadherin in the Sera of Patients With Acute and Persistent Q Fever

**Figure 1:**
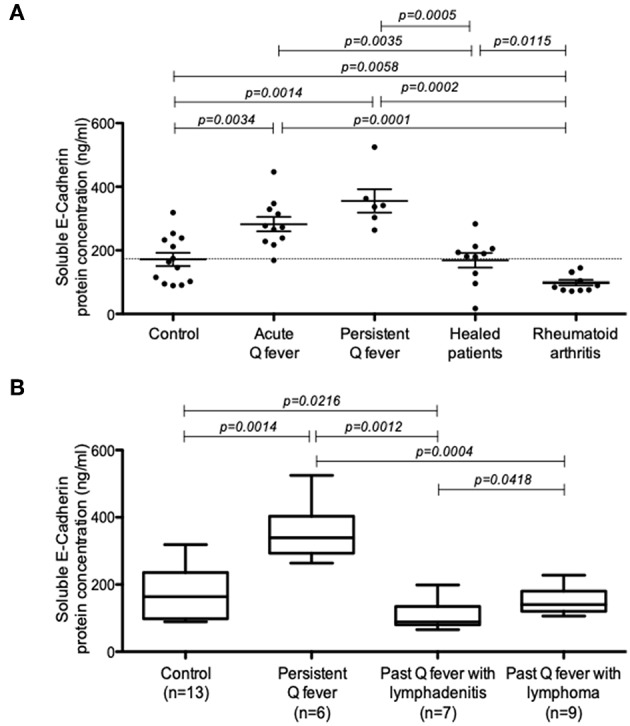
Quantification of sE-cad in different groups of patients. **(A)** ELISA quantification of sE-cad released in the sera of patients with acute Q fever and persistent Q fever compared to control groups consisting in healthy people, people cured of Q fever and a group of patients with rheumatoid arthritis (*p*-values according to Mann–Whitney test). **(B)** ELISA quantification of sE-cad in the sera of patients with persistent Q fever (*n* = 6); and persistent Q fever patients with lymphadenitis (*n* = 7) or lymphoma (*n* = 2 patients and 9 samples for statistical analysis) who were cured from *C. burnetii*.

Soluble E-cadherin (sE-cad), the product of a proteolytic cleavage of cell membrane-anchored E-cad by sheddases, has been described in a few infectious diseases to trigger cancers. Using ELISA, we investigated here the presence of sE-cad in the sera of Q fever patients. As shown in Figure 1A, s-E-cad release was significantly increased in patients with acute Q fever (*n* = 10; *p* = 0.0034) and persistent Q fever (*n* = 6; *p* = 0.0014) compared to the two control groups, the healthy donors' group (*n* = 13), the people cured of Q fever (*n* = 10), and the group of patients suffering from rheumatoid arthritis (*n* = 8). These results indicated that sE-cad cleavage and release were specifically observed in the two forms of Q fever and achieved independently of any inflammatory reaction. We next investigated the serum concentration of sE-cad in the few patients of the cohort who were classified as past persistent focalized Q fever patients but, in addition, suffered from lymphadenitis (*n* = 7) or lymphoma (*n* = 2). The patients carrying lymphomas had been cured from *C. burnetii* for almost 2 and 8 years, respectively. As shown in Figure 1B, the high levels of sE-cad previously found in the sera of acute and persistent Q fever patients were not found for the patients with either lymphadenitis or lymphoma who were considered cured from *C. burnetii*. Altogether, these data indicate that serum sE-cad is high in patients with Q fever.

### Decreased Expression of *CDH1*/E-Cadherin mRNA in Cells From Q Fever Patients

**Figure 2:**
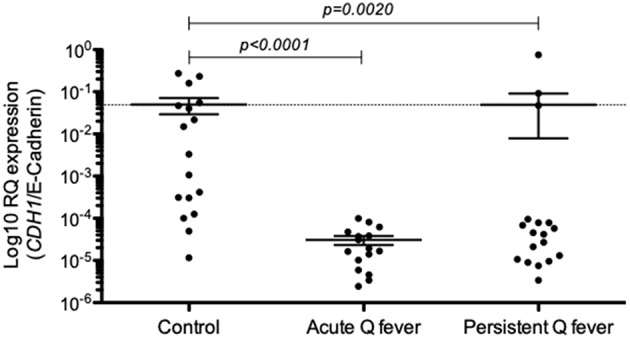
Expression of *CDH1*/E-cadherin in the patients' groups with either acute Q fever (*n* = 15) or persistent Q fever (*n* = 17). mRNA expression was compared to a group of healthy persons (*n* = 17). Data are expressed as log10 RQ were RQ = 2^(−Δ*CT*)^.

Next, we investigated the expression of E-cad mRNA in the PBMCs of both acute and persistent Q fever groups by qRT-PCR. As shown in Figure 2, the expression of E-cad mRNA was significantly down-regulated both in the patients' group with acute Q fever (*n* = 15; *p* < 0.0001) and the patients' group with persistent *C. burnetii* infection (*n* = 17; *p* = 0.002). It is worth noting that the result of E-cad mRNA modulation was more heterogeneous in the persistent Q fever patients' group than in the group of patients with acute Q fever. Indeed, the persistent Q fever group included a few patients showing a level of E-cad mRNA similar to that of the healthy controls. We must also mention a great heterogeneity among the healthy control group in the *CDH1*/E-cadherin gene expression. These results indicated a significantly reduced expression of E-cad mRNA in the PBMCs of acute Q fever and most of the persistent Q fever patients.

### Modulation of Cell-Surface Expression of E-Cadherin in Q Fever Patients' Cell Subpopulations

**Figure 3:**
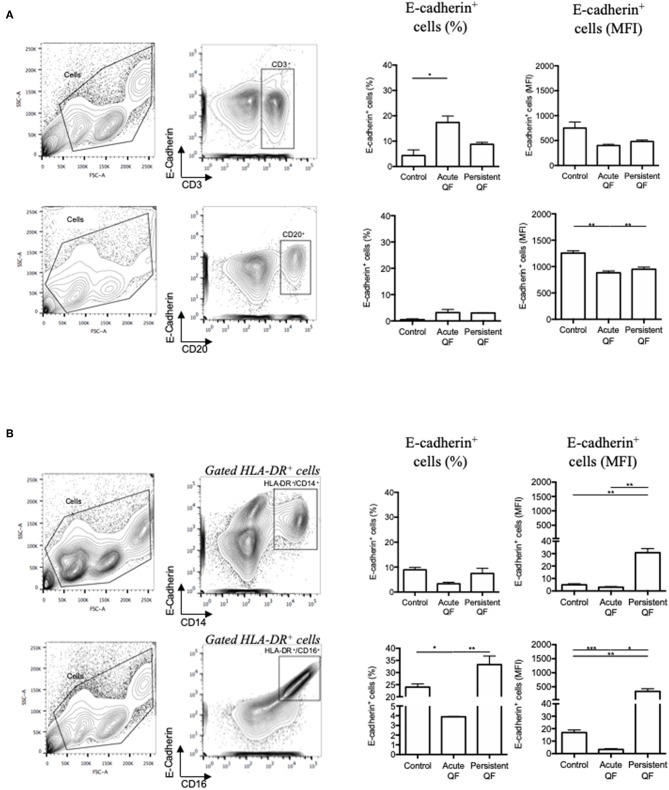
Flow cytometry analysis of E-cadherin expression at the surface of cells from patients with acute Q fever (*n* = 7) and persistent Q fever (*n* = 4) compared to samples from healthy persons (*n* = 13). The gating was performed using different cluster differentiation-specific mAb allowing to select subpopulations of lymphocytes **(A)** CD3^+^ and CD20^+^; **(B)** monocytes HLADR^+^/CD14^+^ and HLADR^+^/CD16^+^. The left panels define the gating chosen; the middle panels show the percent of cells expressing E-cadherin with respect to the cell subpopulations analyzed and the patient's group (healthy donor = control; acute fever patients = Acute QF; and persistent Q fever patients = Persistent QF); the right panels are the histograms of E-cad cell surface expression (MFI) with respect to the cell subpopulations analyzed and the patient's group.

To go further in the understanding of the E-cad expression in Q fever, we measured by flow-cytometry the expression of E-cad in PBMCs subsets isolated from the blood of patients with either acute Q fever or persistent *C. burnetii* infection groups (Figure 3). The flow-cytometry analysis was performed with gating windows on the CD3^+^ (T cells), CD20^+^ (B-cells), and both CD14^+^ and CD16^+^ (monocytes) subpopulations. In the PBMCs from healthy donors, the E-cad was expressed by <5% of the CD3^+^ lymphocytes, <1% of the CD20^+^ lymphocytes, and 9–25% of the HLA-DR^+^ monocytes. A shift in fluorescence intensity for E-cad labeling was observed in some cell subpopulations both in samples from acute (*n* = 7) and persistent focalized Q fever patients (*n* = 4). While E-cad expression at the cell surface of CD3^+^ cells remained similar in patients and controls, we found a moderate but significant decrease of E-cad expression that concerns about 3% of the CD20^+^ cells during both acute (*p* = 0.0012) and persistent focalized (*p* = 0.0044) Q fever. Although, cell-surface expression of E-cad remained stable in HLADR^+^/CD14^+^ cells, or significantly decreased in HLADR^+^/CD16^+^ cells from acute Q fever patients (*p* = 0.0004), it was significantly increased in both the HLADR^+^/CD14^+^ (*p* = 0.0039) and HLADR^+^/CD16^+^ (*p* = 0.0039) cell subpopulations from persistent Q fever patients. A greater over-expression of cell surface E-cad was found within more than 30% of the HLADR^+^/CD16^+^ subpopulation. At the level of cell subpopulations, the reduction of E-cad protein expression in B-cells corroborates the decrease in E-cad mRNA observed with PBMCs of Q fever patients. Despite the significant global decrease in E-cad mRNA observed with the PBMCs of both acute and persistent Q fever patients, we found a surprising over-expression of cell-surface E-cad protein on the HLADR^+^/CD16^+^ cells of persistent Q fever patients.

### Differential Expression of *CTNNB1*/β-Catenin mRNA Between Acute and Persistent Q Fever Patients

**Figure 4:**
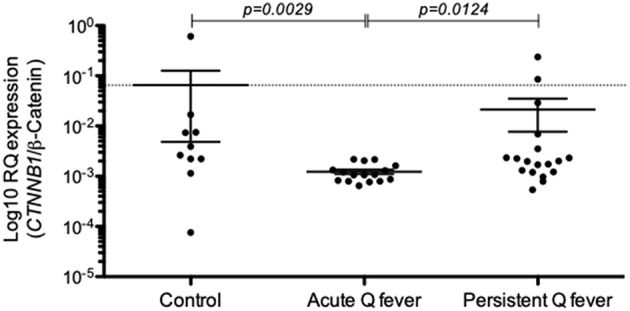
Expression of *CTNNB1*/β-catenin mRNAs in the patients' groups with either acute Q fever (*n* = 15) or persistent Q fever (*n* = 17). mRNA expression was compared to a group of healthy persons (*n* = 10). Data are expressed as log10 RQ were RQ = 2^(−Δ*CT*)^.

Because E-Cad protein expressed at the cell membrane is expected to bind β-cat through its intracytoplasmic tail and thus regulate cell signaling, we investigated whether or not Q fever is associated with a modulation of β-cat mRNA expression. We found that the expression of *CTNNB1* mRNA coding for β-cat in PBMCs of Q fever patients was significantly reduced in acute Q fever (*n* = 15; *p* = 0.0029) compared to healthy controls and patients with persistent Q fever (Figure 4). The β-cat mRNA modulation was more heterogeneous in the persistent Q fever patients' group (*n* = 17); within this group, there was no statistically significant difference with the healthy control group. These results indicate that β-cat gene expression is down-modulated during the acute phase of Q fever, yet its expression may then vary in patients who suffer from persistent Q fever.

### Modulation of *CDH-1* Gene Expression and Membrane E-cad in PBMCs Exposed *in vitro* to *C. burnetii* Infection

**Figure 5:**
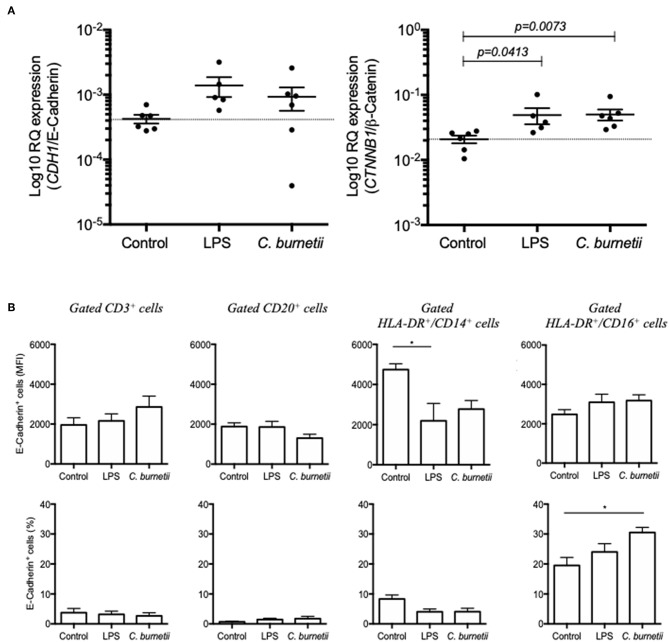
**(A)** qRT-PCR analysis of *CDH1*/E-cadherin (left panel) and *CTNNB1*/β-catenin (right panel) mRNAs expression in PBMCs from six healthy donors exposed *in vitro* during 8 h to *C. burnetii* Nine Mile strain. The gene expression is compared to that of PBMCs maintained germ-free (control) and PBMCs cultured in medium supplemented with LPS from *E. coli*. **(B)** Flow cytometry analysis of E-cad expression at the surface of PBMCs (from six different donors) exposed *in vitro* for 8 h to live *C. burnetii*. The gating was performed using different cluster differentiation-specific mAb allowing to select subpopulations of lymphocytes: CD3^+^, CD20^+^, and monocytes HLADR^+^/CD14^+^ and HLADR^+^/CD16^+^.

In order to evidence a direct relationship between the modulation of E-cad expression and the presence of *C. burnetii* in the culture medium of human cells, PBMCs from six healthy donors were exposed *in vitro* during 8 h to 10^7^ live *C. burnetii* Nine Mile (RSA493) reference strain. The modulation of *CDH1*/E-cadherin mRNA and *CTNNB1*/β-catenin mRNA was analyzed by qRT-PCR. As shown in Figure 5A, there was a tendency for over-expression of *CDH1*/E-cadherin mRNA in PBMCs exposed to *C. burnetii* but it was not statistically significant. A significant (*p* = 0.0073) over-expression of *CTNNB1*/β-catenin mRNA was found for PBMCs exposed to *C. burnetii*. It is worth noting that the same tendency for over-expression of *CDH1*/E-cadherin mRNA was observed after PBMCs had been exposed to LPS from *E. coli* and a significant (*p* = 0.0413) over-expression of *CTNNB1*/β-catenin mRNA was found under these experimental conditions.

In order to determine the cell subpopulation(s) on which *C. burnetii* might act on cellular genes transcription to modulate the expression of E-cad, PBMCs were fixed 8 h after incubation with *C. burnetii* Nine Mile strain and analyzed by flow-cytometry for E-cad surface expression. As shown in Figure 5B, a significant increase in the percentage of HLA-DR+/CD16+ monocytes expressing E-cad at their surface was measured. This result corroborates the data got with cell subpopulations isolated from persistent Q fever patients.

## Discussion

We report here the first experimental evidence that sE-cad is detected in the sera of patients with acute or persistent Q fever, indicating that sE-cad can be considered as a new biomarker of Q fever. The release of sE-cad is accompanied by modifications in the transcription of the *CDH1* gene and by modulation of the E-cad expression at the surface of cells.

Increased serum levels of sE-cad have been reported in a number of metabolic and inflammatory diseases as well as cancers (Grabowska and Day, 2012). Moreover, a role for sE-cad as tumorogenic co-factor was highlighted in the infections by *H. pylori, B. fragilis*, and *S. gallolyticus*, that trigger gastric adenocarcinoma or colorectal tumors (Wu et al., 2003; O'Connor et al., 2011; Kumar et al., 2017; Chung et al., 2018). Herein, we demonstrate that sE-cad is increased in Q fever but fail to find an association between sE-cad release and the induction of NHL. Our data strongly suggest that the presence of sE-cad in the sera of Q fever patients is a marker of a metabolic disorder and/or bacterial invasion. Measurement of sE-cad could advantageously be added to the set of biological parameters aimed at monitoring patients with Q fever. Interestingly, sE-cad is no longer found increased in the sera of patients who had been considered cured of *C. burnetii* (people who lack clinical symptoms but show elevated phase I IgG). These observations suggest that sE-cad release might require the presence of *C. burnetii*-infected cells circulating in the patient. Working on patients' cell subpopulations, we found a significant decreased of E-cad that concerns a small percentage (<5%) of the CD20^+^ B-cells subpopulation. In the *in vitro* infection assay, we also observed the same tendency for the CD20^+^ B-cells. This E-cad down-regulation could either be due to sheddase activation or *CDH1* gene repression in B-cell infected by *C. burnetii*, continuous exposure of B cells to *C. burnetii* lipopolysaccharides, or aberrant bystander cell signaling in their microenvironment (Amano et al., 1987; Lee, 1993; Melenotte et al., 2016). Heterotypic binding of E-cad on epithelial tissues to CD103 on lymphocytes is well-documented. In contrast the functional role of E-cad on the surface of lymphocytes has not yet been addressed *in vivo*. Although the expression of E-cad on subpopulations of mature human lymphocytes has not been the subject of intense research until now because this molecule is usually absent from mature lymphocytes, it is likely that the differential expression of these molecules under pathological conditions reflects changes in the transmigration and homing capacity of these cells (Reyat et al., 2017). Conversely, we found that the E-cad protein expression at the surface of the cells of patients with Q fever was significantly increased on both CD14^+^ and CD16^+^ monocytes from persistent Q fever patients. We observed a greater expression of E-cad that concerns more than 30% of CD16^+^ monocytes, a cell subpopulation that we previously demonstrated as being a target for *C. burnetii* in humans (Ka et al., 2016). In addition, we found that *C. burnetii* infection triggers a reduced expression of E-cad mRNA in the PBMCs of acute Q fever patients. Although sE-cad release in sera and decreased E-cad mRNA expression in PBMCs was also observed in the persistent Q fever patients' group, it is clear that there are marked differences between individuals within this second group, and their mean level of E-cad mRNA was found similar to that of the healthy controls. Moreover, in contrast to the data obtained with cell subpopulations of acute Q fever patients, high cell surface expression of E-cad was found in both CD14^+^ and CD16^+^ cell subpopulations from persistent Q fever patients. When PBMCs from healthy donors were exposed *in vitro* to *C. burnetii* Nine Mile strain for 8 h and analyzed by flow cytometry, about 30% of the CD16+ monocytes were found to express cell-surface E-cad. This increase in the percentage of cells expressing cell-surface E-cad might be either due to *C. burnetii* polysaccharide induced signaling after cell-surface binding or transduction of signal by intracellular live bacteria. *In vitro* infection studies by Heinzen and collaborators have shown that the avirulent strain of *C. burnetii* induced DCs maturation, whereas cell maturation was not observed with the Nine Mile virulent strain of *C. burnetii* (Shannon et al., 2005; Larson and Heinzen, 2017).

Although rare, the incidence of NHL B-lymphoma in patients infected by *C. burnetii* was significantly higher (25-fold) than within the general French population (Melenotte et al., 2016). Despite intensive work to identify the bacterial compounds supporting *C. burnetii* replication and virulence (Deng et al., 2012; Shipman et al., 2013; Stead et al., 2013; Martinez et al., 2014, 2016), the molecular mechanism involved in *C. burnetii*-induced Q fever and NHL-associated to Q fever remains largely unknown. Interestingly, E-cad is known to act as a tumor-suppressing protein which inhibits the canonical Wnt-Frizzled signaling pathway by sequestration of β-Cat. Besides, the aberrant expression of E-cad can contribute to cellular transformation through β-Cat dysregulation leading to activation of β-Cat /T-cell factor signaling (Gottardi et al., 2001; Marambaud, 2002; Gall and Frampton, 2013; Kourtidis et al., 2013). The shedding of sE-cad can also allow extracellular domains of E-cad interaction with the ErbB receptors inducing their transactivation (Zheng et al., 2009). Therefore, we speculate that the release of sE-cad might participate to the molecular crosstalk which takes place in the microenvironment of the lymph node during persistent Q fever and might possibly trigger a pro-carcinogenic program required for the initiation of NHL lymphoma. In this hypothesis, one should expect that E-cad cleavage would allow β-cat translocation to the nucleus for trans-activation of proliferative genes. Surprisingly, we observed a down-regulation in β-cat mRNA expression in PBMCs of acute and persistent Q fever patients, which does not seem support this model and remains to be explained. Yet, when PBMCs were exposed *in vitro* to *C. burnetii*, a significant over-expression of *CTNNB1*/β-catenin mRNA was measured, and this phenomenon should be further explored. In Q fever, overproduction of interleukin-10 (IL-10) by infected monocytes was found critical in both sustaining replication of the *C. burnetii* and preventing the macrophages microbicidal activity (Mege et al., 2006; Ka et al., 2014). High levels of this B-cell growth factor are known to increase BCL-2 expression in lymphoma cells (Voorzanger et al., 1996), and to correlate with a worse prognosis in DLBCL patients (Lech-Maranda et al., 2004). The Rituximab used in the treatment of NHL patients may overcome BCL-2-mediated chemoresistance through the inhibition of IL-10, thus down-regulating BCL-2 expression (Park et al., 2009). By investigating a specific gene signature for Q fever patients by microarray, we recently found that specific genes involved in anti-apoptotic process were over-expressed compared to samples from healthy donors, and that pro-apoptotic genes were repressed (Melenotte et al., 2019).

Another interesting question concerns the molecular mechanism by which cleavage of E-cad is achieved (Niessen et al., 2011). It is likely that E-cad underwent a catalytic cleavage by a protease of human origin during the acute and persistent phases of the disease. Yet, we cannot currently exclude that a sheddase of bacterial origin could be produced by *C. burnetii*. In the literature the proteolytic cleavage of E-cad was reported for a number of sheddases, including zinc-dependent matrix metalloproteinases (MMP-2, -3, -7, -9 and -14), members of the disintegrin family (adamalysin/ADAM-10 and−15), bacterial proteases gingipains (HRgpA, RgpB, and Kgp), *B. fragilis* toxin/fragilysin, cysteine cathepsins (B, L, and S), serine protease Kallikrein-7 (KLK7), plasmin serine protease, aspartic proteinases BACE1 and BACE2, and malaria parasite serine proteinases PfSUB2n, PfROM1, and PfROM 4 (Grabowska and Day, 2012). One possible candidate for E-cad cleavage during *C. burnetii* infections could be the human MMP-9, since: (i) MMP-9 levels were reported elevated in the sera of patients with endocarditis (Thuny et al., 2012), and patients with acute Q fever (Krajinović et al., 2012); (ii) its production was induced in PBMCs of healthy persons following *in vitro* exposure to *C. burnetii*; (iii) MMP-9 SNP was found more frequently in patients with persistent Q fever (Jansen et al., 2017); and (iv) MMP-9 was recently identified as a key gene in mantle cell lymphoma (Yan et al., 2018). Experiments are under progress in our laboratory to investigate whether *C. burnetii* infection induces the activation of a human cellular protease from the sheddase family or if *C. burnetii* itself encodes for a sheddase.

In conclusion, this work describes for the first time the modulation of the E-cad signaling pathway and release of sE-cad in the sera of Q fever patients. It opens a new avenue of research to understand the pathophysiology of Q fever and the role played by E-cad during *C. burnetii* invasion of human tissues.

## Data Availability

The raw data supporting the conclusions of this manuscript will be made available by the authors, without undue reservation, to any qualified researcher.

## Author Contributions

SM, IO, and CS performed the experiments. CS and CM analyzed clinical data. SM and CC performed the flow cytometry analysis. CD supervised the experimental work. J-LM and DR supervised the clinical studies. SM, J-LM, and CD conceived and wrote the paper.

### Conflict of Interest Statement

The authors declare that the research was conducted in the absence of any commercial or financial relationships that could be construed as a potential conflict of interest.
